# Supplementary material for: The role of implementation climate in shaping early essential newborn care practice: Insights from a multi-center cross-sectional study in China
Source: PLoS One. 2025 Oct 17;20(10):e0334855. doi: 10.1371/journal.pone.0334855 (PMC12533878; doi:10.1371/journal.pone.0334855)
Supplement: S1 File — (DOCX) [file pone.0334855.s001.docx]

**Supplement 1 Questionnaire**

**Part 1 Individual characteristics**

1.1How old are you?

_________________________________

1.2What is your gender?

| ○Female |
| --- |
| ○Male |

1.3What is your parental status?

| ○without child |
| --- |
| ○with child |

1.4 What is your current education?

| ○vocational |
| --- |
| ○bachelor |
| ○master |
| ○doctorate or upper |

1.5. Do you have position?

| ○Nurse supervisor |
| --- |
| ○suboptimal leader |
| ○no |
| ○other： _________________ |

1.6. Have you received training specifically in basic Early Neonatal Care Technologies (EENC)?

| ○yes |
| --- |
| ○no |

1.7. How many years have you work in delivering room?

1.8. Does your hospital have routine workflow on EENC?

| ○yes |
| --- |
| ○no |

1.9. How heavy is your current workload?

| ○heavy |
| --- |
| ○acceptable |

**Part 2 Nurse-midwives’ practice of EENC**

2.1 Place a normal newborn on the mother's abdomen, dry and stimulate the newborn within 30 seconds.

| ○never |
| --- |
| ○seldom |
| ○often |
| ○always |

2.2 Routine suction of newborn mouth and nose.

| ○never |
| --- |
| ○seldom |
| ○often |
| ○always |

2.3 Normally, the umbilicus is clamped immediately after birth.

| ○never |
| --- |
| ○seldom |
| ○often |
| ○always |

2.4 If the newborn is in good condition, do not separate the newborn from the mother, keep the newborn naked and in contact with the mother's skin, and cover with a towel.

| ○never |
| --- |
| ○seldom |
| ○often |
| ○always |

2.5 The broken end of the neonatal umbilical cord is routinely disinfected.

| ○never |
| --- |
| ○seldom |
| ○often |
| ○always |

2.6 Keep the newborn in skin-to-skin contact with the mother for 90 minutes without special circumstances.

| ○never |
| --- |
| ○seldom |
| ○often |
| ○always |

2.7 Encourage and help the mother breastfeeding.

| ○never |
| --- |
| ○seldom |
| ○often |
| ○always |

2.8 Routine care (physical examination, body measurement, vaccination, etc.) is performed 60-90 minutes after skin contact.

| ○never |
| --- |
| ○seldom |
| ○often |
| ○always |

2.9 Neonatal eye care is routinely given to prevent infection, such as erythromycin.

| ○never |
| --- |
| ○seldom |
| ○often |
| ○always |

2.10 The newborn is routinely given 1mg intramuscular injection of vitamin K

| ○never |
| --- |
| ○seldom |
| ○often |
| ○always |

**Part 3 Nurse-midwives’ knowledge of EENC**

3.1Do you think it is normal for newborns to be dried immediately after birth?

| ○yes |
| --- |
| ○no |
| ○not sure |

3.2Do you think it is necessary for the newborn to have a regular suction nose and mouth after delivery?

3.3 Do you think not being able to suck or cry is a red alarm for a newborn?

| ○yes |
| --- |
| ○no |
| ○not sure |

3.4Do you think that under normal circumstances, should the umbilical cord be severed immediately after birth?

| ○yes |
| --- |
| ○no |
| ○not sure |

3.5 Do you think a broken umbilical cord of a newborn should be routinely disinfected?

| ○yes |
| --- |
| ○no |
| ○not sure |

3.6 Do you think that under normal circumstances, a newborn should have skin-to-skin contact with its mother immediately after birth?

| ○yes |
| --- |
| ○no |
| ○not sure |

3.7Do you think it is normal for newborns to have uninterrupted skin contact with their mothers for up to 90 minutes?

| ○yes |
| --- |
| ○no |
| ○not sure |

3.8Do you think newborn eye care should be routinely given to prevent infection?

| ○yes |
| --- |
| ○no |
| ○not sure |

3.9 Do you think 1mg of vitamin K should be routinely given to newborns by intramuscular injection?

| ○yes |
| --- |
| ○no |
| ○not sure |

**Part 4 Nurse-midwives’ attitude towards EENC**

*0 means "completely disagree", 1 means "somewhat agree", 2 means "somewhat agree", 3 means "strongly agree" and 4 means "completely agree".*

4.1 I agree with the positive clinical effects of early skin-to-skin contact between mother and child

|  | ○0 | ○1 | ○2 | ○3 | ○4 |
| --- | --- | --- | --- | --- | --- |

4.2 I agree with the positive clinical effectiveness of early exclusive breastfeeding

|  | ○0 | ○1 | ○2 | ○3 | ○4 |
| --- | --- | --- | --- | --- | --- |

4.3 I agree with the positive clinical effectiveness of delayed cord disconnection

|  | ○0 | ○1 | ○2 | ○3 | ○4 |
| --- | --- | --- | --- | --- | --- |

4.4 I agree with the safety of non-routine disinfection of the broken cord

|  | ○0 | ○1 | ○2 | ○3 | ○4 |  |
| --- | --- | --- | --- | --- | --- | --- |

4.5 I agree with the safety of not conventionally engaging the mouth and nose

|  | ○0 | ○1 | ○2 | ○3 | ○4 |
| --- | --- | --- | --- | --- | --- |

4.6 I am positive about the development prospects of EENC technology

|  | ○0 | ○1 | ○2 | ○3 | ○4 |
| --- | --- | --- | --- | --- | --- |

4.7 I think the implementation of EENC will give us a greater sense of achievement

|  | ○0 | ○1 | ○2 | ○3 | ○4 |  |
| --- | --- | --- | --- | --- | --- | --- |

4.8 I think I have the ability to master the EENC technology

|  | ○0 | ○1 | ○2 | ○3 | ○4 |  |
| --- | --- | --- | --- | --- | --- | --- |

4.9 I think the implementation of EENC is helpful to the health of mother and child

|  | ○0 | ○1 | ○2 | ○3 | ○4 |  |
| --- | --- | --- | --- | --- | --- | --- |

4.10I think if we don't implement the EENC, our hospital will fall behind other hospitals in terms of midwifery services.

|  | ○0 | ○1 | ○2 | ○3 | ○4 |  |
| --- | --- | --- | --- | --- | --- | --- |

**Part 5 The implementation climate in organizations**

Please make your choice based on how well you think your team's evidence-based practice is working and how well it matches the statements below. Please select a value based on your level of agreement with the following statements: 0 indicates "completely disagree", 1 indicates "somewhat agree", 2 indicates "somewhat agree", 3 indicates "strongly agree", and 5 indicates "completely agree".

1.One of the main goals of our team's clinical work is to effectively carry out evidence-based practice.

|  | ○0 | ○1 | ○2 | ○3 | ○4 |  |
| --- | --- | --- | --- | --- | --- | --- |

2. Members in our team believe it is important to develop evidence-based practice.

|  | ○0 | ○1 | ○2 | ○3 | ○4 |  |
| --- | --- | --- | --- | --- | --- | --- |

3. Developing evidence-based practice is a top priority for our team/institutional care

|  | ○0 | ○1 | ○2 | ○3 | ○4 |  |
| --- | --- | --- | --- | --- | --- | --- |

4. Our team/organization offers conferences, workshops or seminars for nurses that focus on evidence-based practice.

|  | ○0 | ○1 | ○2 | ○3 | ○4 |  |
| --- | --- | --- | --- | --- | --- | --- |

5. Our team provides nurses with evidence-based practical training or on-the-job training opportunities.

|  | ○0 | ○1 | ○2 | ○3 | ○4 |  |
| --- | --- | --- | --- | --- | --- | --- |

6. Our team/organization provides nurses with learning materials, journals, etc. for evidence-based practice.

|  | ○0 | ○1 | ○2 | ○3 | ○4 |  |
| --- | --- | --- | --- | --- | --- | --- |

7. In our team/organization, nurses who carry out evidence-based practice in their clinical work are considered clinical experts.

|  | ○0 | ○1 | ○2 | ○3 | ○4 |  |
| --- | --- | --- | --- | --- | --- | --- |

8. In our team/institution, nurses who carry out evidence-based practice in their clinical work are highly respected.

|  | ○0 | ○1 | ○2 | ○3 | ○4 |  |
| --- | --- | --- | --- | --- | --- | --- |

9. In our team/organization, nurses who develop evidence-based practices in their clinical work are more likely to be promoted.

|  | ○0 | ○1 | ○2 | ○3 | ○4 |  |
| --- | --- | --- | --- | --- | --- | --- |

10. Our team/organization provides financial support to advance evidence-based practice.

|  | ○0 | ○1 | ○2 | ○3 | ○4 |  |
| --- | --- | --- | --- | --- | --- | --- |

11. In our team/institution, nurses who are more capable of evidence-based practice are more likely to be rewarded.

|  | ○0 | ○1 | ○2 | ○3 | ○4 |  |
| --- | --- | --- | --- | --- | --- | --- |

12. Our team/organization is compensated for time spent participating in evidence-based practice-related projects or training.

|  | ○0 | ○1 | ○2 | ○3 | ○4 |  |
| --- | --- | --- | --- | --- | --- | --- |

13. When selecting members for the evidence-based Practice program, our team/institution tends to select nurses who have conducted evidence-based practice before.

|  | ○0 | ○1 | ○2 | ○3 | ○4 |  |
| --- | --- | --- | --- | --- | --- | --- |

14. When selecting members for the evidence-based Practice program, our team/institution prefers to select nurses who have been trained in systematic evidence-based practice.

|  | ○0 | ○1 | ○2 | ○3 | ○4 |  |
| --- | --- | --- | --- | --- | --- | --- |

15. When selecting members for the evidence-based Practice program, our team/institution tends to select nurses who value evidence-based practice.

|  | ○0 | ○1 | ○2 | ○3 | ○4 |  |
| --- | --- | --- | --- | --- | --- | --- |

16. When selecting members for the evidence-based practice project, our team tends to choose nurses who are adaptable.

|  | ○0 | ○1 | ○2 | ○3 | ○4 |  |
| --- | --- | --- | --- | --- | --- | --- |

17. When selecting members for the evidence-based Practice program, our team/institution prefers nurses who are flexible.

|  | ○0 | ○1 | ○2 | ○3 | ○4 |  |
| --- | --- | --- | --- | --- | --- | --- |

18. When selecting members for the evidence-based Practice program, our team/institution tends to select nurses who are open to new ways of practicing.

|  | ○0 | ○1 | ○2 | ○3 | ○4 |  |
| --- | --- | --- | --- | --- | --- | --- |
