# Supplementary material for: The role of implementation climate in shaping early essential newborn care practice: Insights from a multi-center cross-sectional study in China
Source: PLoS One. 2025 Oct 17;20(10):e0334855. doi: 10.1371/journal.pone.0334855 (PMC12533878; doi:10.1371/journal.pone.0334855)
Supplement: S2 File — (DOCX) [file pone.0334855.s002.docx]

**Supplement 2. The knowledge, attitude and practice of EENC**

| **Knowledge: Do you think the following interventions are needed during the delivery?** | **Frequency n (%)** | |  |
| --- | --- | --- | --- |
|  | **Correct** | **Incorrect** |  |
| 1. Dry immediately | 411(94.9) | 22(5.1) |  |
| 2. Routine suction of newborn’s mouth and nose | 359(82.9) | 74(17.1) |  |
| 3. Inability to suckle or cry is a red flag for a newborn | 276(63.7) | 157(36.3) |  |
| 4. Clamp the umbilical cord immediately after birth | 423(97.7) | 10(2.3) |  |
| 5. Disinfect the umbilical ending routinely | 228(52.7) | 205(47.3) |  |
| 6. Keep the baby skin-to-skin contact with mother immediately after birth | 426(98.4) | 7(1.6) |  |
| 7. Maintain skin-to-skin contact between baby and mother for 90 minutes | 401(92.6) | 32(7.4) |  |
| 8. Give routine eye care to baby to prevent infection (e.g., erythromycin) | 125(28.9) | 308(71.1) |  |
| 9. Give baby 1 mg intramuscular injection of vitamin K | 343(79.2) | 90(20.8) |  |
| **Attitude: To what extend do you agree with the following statements?** | **Mean (SD)** | |  |
| 1. I recognize the positive clinical effects of early skin-to-skin contact between babies and mothers | 3.81(±0.52) | |  |
| 2. I recognize the positive clinical effects of early initiation of breastfeeding | 3.82(±0.50) | |  |
| 3. I recognize the positive clinical effects of delaying clamping the umbilical cord | 3.76(±0.57) | |  |
| 4. I recognize the safety of not disinfecting the umbilical cord ending routinely | 2.97(±1.23) | |  |
| 5. I recognize the safety of not sucking the nose and mouth of babies routinely | 3.39(±0.95) | |  |
| 6. I hold a positive attitude towards the development of EENC technology | 3.77(±0.52) | |  |
| 7. I think implementing EENC will give us a greater sense of achievement | 3.55(±0.74) | |  |
| 8. I think I have the ability to master the knowledge and practice of the EENC | 3.64(±0.61) | |  |
| 9. I think the implementation of EENC could benefit mothers and babies | 3.73 (±0.55) | |  |
| 10. I think if we don't implement EENC, our hospital will fall behind other hospitals in terms of midwifery services | 3.13(±1.10) | |  |
| **Practice: In your daily work, do you include the following interventions?** | **Frequency n (%)** | |  |
|  | **Correct** | **Incorrect** |  |
| 1. Dry the baby immediately after birth | 411(94.9) | 22(5.1) |  |
| 2. No routine suction of newborn’s mouth and nose | 334(77.1) | 99(22.9) |  |
| 3. No clamping of the umbilical cord immediately after birth | 399(92.1) | 34(7.9) |  |
| 4. Maintain skin-to-skin contact between baby and mother | 383(88.5) | 50(11.5) |  |
| 5. No disinfecting of the umbilical ending | 194(44.8) | 239(55.2) |  |
| 6. Maintain skin-to-skin contact between baby and mother for 90 minutes | 374(86.4) | 59(13.6) |  |
| 7. Encourage and instruct mothers in the early initiation of breastfeeding | 429(99.1) | 4(0.9) |  |
| 8. Routine care (physical examination, body measurement, vaccination, etc.) is administered after skin-to-skin contact | 348(80.4) | 85(19.6) |  |
| 9. Give routine eye care to baby to prevent infection (e.g., erythromycin) | 187(43.2) | 246(56.8) |  |
| 10. Give baby 1 mg intramuscular injection of vitamin K | 385(88.9) | 48(11.1) |  |

EENC: early essential newborn care
