# Supplementary material for: The role of implementation climate in shaping early essential newborn care practice: Insights from a multi-center cross-sectional study in China
Source: PLoS One. 2025 Oct 17;20(10):e0334855. doi: 10.1371/journal.pone.0334855 (PMC12533878; doi:10.1371/journal.pone.0334855)
Supplement: Attachment — Submitted filename: reviewers comments.pdf [file pone.0334855.s003.pdf]

Dear Editors and Authors,

Thank you for giving me the opportunity to review the manuscript, under the title "The Role of Implementation Climate in Shaping Early Essential Newborn Care (EENC) Practice: Insights from a Multi-Center Cross-Sectional Study in China".

The authors chose a very important topic on newborn care in China. This study was conducted between Dec 2022 and April 2023 among nurse-midwives from 12 tertiary-level maternity facilities across China. The authors developed and validated a structured questionnaire, using the WHO guidelines. The study examined EENC-related knowledge, attitudes, practices, and perceived implementation climate, using path analysis and logistic regression to assess direct and indirect associations. The authors reported prevalence of good EENC practice, mean score of implementation climate, and found that good knowledge, positive attitudes, training, leadership role, and perceived workload to be predictors of good EENC practice (with heavier workloads having a negative effect on positive attitude, which impacted good EENC practice, and with good knowledge, in-service training, positive attitude, and leadership role having a positive effect on good EENC practice).

Better implementation climate had positive effects on attitude, and implementation climate positively affected EENC practice indirectly through attitudes.

The authors concluded that both individual factors and organizational climate are important influential factors of EENC practices. To improve EENC implementation, healthcare systems should focus on enhancing the implementation climate through leadership support, training staff, appropriate reward systems, and addressing workload challenges.

My overall assessment of the manuscript is that it has been written well and can be considered for publication. There are some minor issues that the authors need to address.

1. In table3 and table 4, there is an inconsistency in using "bivariable" and "univariable". The authors can use either one; but this should be consistent throughout the manuscript.
2. For figure1, some words start with capital letters; while others start with small letters (e.g., Training, knowledge). They need to be consistent – either start with small or capital letters.
3. Oversampling of female nurse-midwives can be a potential limitation in this study. The authors may opt to discuss it in the limitation of this study, because male nurses may have different attitudes and practices with respect to EENC, although I understand that midwives are female and this sample can present a valid representation of those health workers, who are mainly involved in newborn care.
