# Supplementary material for: The role of implementation climate in shaping early essential newborn care practice: Insights from a multi-center cross-sectional study in China
Source: PLoS One. 2025 Oct 17;20(10):e0334855. doi: 10.1371/journal.pone.0334855 (PMC12533878; doi:10.1371/journal.pone.0334855)
Supplement: Attachment — Submitted filename: Response to Reviewers.docx [file pone.0334855.s005.docx]

**Response letter**

Dear Editors and Reviewers:

Thank you for your letter and for the time you have taken to review our manuscript. We greatly appreciate your valuable feedback, which has been essential in improving our paper. We have carefully studied all comments and have incorporated revision accordingly. The main corrections and our point-by point response are provided below.

**Reviewer 1**

**Abstract: it is better to remove bullet numbering in your results section: (1)..., (2)...., (3).... please write your results in continues prose.**

**Response:** We thank you for this suggestion. The results section of the abstract has been revised into continuous prose by removing the bullet points, which improves the flow and readability.

**Methodology: your tools for measurement of attitude was developed by authors? if yes please give more information about its validity.**

**Response:** Thank you for raising this important point. Yes, the attitude measurement tool was developed by the authors. Its validity was supported through a combination of qualitative insight, expert review, and statistical evaluation.

The tool was designed based on qualitative fieldwork, expert review, and psychometric testing. Specifically, we included items 4 and 5 to capture locally relevant attitudes toward two controversial EENC practices—non-routine cord disinfection and non-traditional suctioning—which were frequently raised by midwives in our context. Although Items 4, 5, and 10 had modest factor loading, they were retained due to their contextual and theoretical relevance. Item 10, in particular, reflects institutional motivation and perceived competitive pressure, a dimension not covered by other items.

Except for these three items, all other items exhibited standardized regression weights ranging from 0.596 to 0.905, exceeding the conventional threshold of 0.5, thus indicating satisfactory convergent validity for the latent construct of attitude.

Below are the standardized regression weights for all items:

|  | Estimate |
| --- | --- |
| Item 1<---Attitude | 0.891 |
| Item 2<---Attitude | 0.898 |
| Item 3<---Attitude | 0.834 |
| Item 4<---Attitude | 0.253 |
| Item 5<---Attitude | 0.414 |
| Item 6<---Attitude | 0.905 |
| Item 7<---Attitude | 0.629 |
| Item 8<---Attitude | 0.596 |
| Item 9<---Attitude | 0.687 |
| Item 10<---Attitude | 0.322 |

We hope this clarification adequately addresses your question regarding the validity of the attitude scale.The corresponding modifications have been incorporated into the revised manuscript on line 164-173.

**Results: Table 3, 4: COR and AOR; clarify abbreviations at first mention, and please write ratio instead of radio.**

**Response:**Thank you for these valuable corrections. We have clarified the abbreviations "COR" (Crude Odds Ratio) and "AOR" (Adjusted Odds Ratio) at their first mention in the text (Line 282 and Line 200, respectively). Additionally, the typographical error of "radio" has been corrected to "ratio".

**Reviewer 2**

**In table3 and table 4, there is an inconsistency in using “bivariable” and“univariable”. The authors can use either one; but this should be consistent throughout the manuscript.**

**Response:**We sincerely thank the reviewer for this careful observation and valuable comment. We apologize for this inconsistency in our terminology.We have now revised the manuscript to consistently use the term "univariable" throughout, including in Tables 3 and 4 and in the corresponding descriptions in the main text.

**2. For figure1, some words start with capital letters; while others start with small letters (e.g., Training, knowledge). They need to be consistent – either start with small or capital letters.**

**Response:**Thank you for this helpful feedback. We have revised Figure 1 to ensure consistent capitalization (all words now begin with a capital letter) and have re-uploaded the updated figure.

**3. Oversampling of female nurse-midwives can be a potential limitation in this study. The authors may opt to discuss it in the limitation of this study, because male nurses may have different attitudes and practices with respect to EENC, although I understand that midwives are female and this sample can present a valid representation of those health workers, who are mainly involved in newborn care.**

**Response:**We thank you for this insightful comment. We agree that the predominance of female nurse-midwives in our sample is an important point to address. Although this reflects the real-world gender distribution of midwifery professionals in the study context—where midwives are predominantly female—we acknowledge that including more male nurses could provide additional perspectives on EENC attitudes and practices. We have added this point to the limitations section on line 425-429 to offer a more comprehensive discussion of the generalizability of our findings.

**Additional Editor Comments**

**Abstract: It is better to write results section paragraph-vise rather to present in a bullet manner.**

**Response:**Thank you for the suggestion. We have revised the results section of the abstract into a paragraph format as recommended.

**The authors should proof read the entire article for language correction and grammar.**

**Response:**Thank you for this comment.Our team has carefully re-examined the manuscript to address grammatical issues and refine the expression. We would be grateful if the reviewer could point out any specific passages that still require attention.

**Please look at published papers on PLOS ONE for in-text citation. The authors should a single space between the in-text citation and text. In most cases, they are now attached.**

**Response:**Thank you for this meticulous feedback. We have carefully reviewed the entire manuscript and ensured that a single space is now consistently applied between all in-text citations and the preceding text.

**Tables 3 & 4: Radio should be ratio.**

**Response:** We thank the reviewer for pointing out this typographical error. We have corrected "Radio" to "Ratio" in both Table 3 and Table 4. We apologize for any confusion this may have caused.

**Line 104: Settings should be with small letter.**

**Response:**Thank you for pointing this out. The word on line 104 has been corrected to "settings" as suggested. We have also taken care to ensure consistent capitalization throughout the manuscript.

**Discussion: Please make only the first letter of subheading capital.**

**Response:** Thank you for pointing this out. We have revised the subheading in the Discussion section to have only the first letter capitalized.

**Line 337-339: Add the following citations** <https://journals.plos.org/plosone/article?id=10.1371/journal.pone.0324758>

**Response:** We agree with your suggestion. The recommended citation has been added to the discussion section (lines 343–345), as it provides valuable external validation for our argument on the necessity of ongoing training.

The added sentence reads: “This is consistent with another study from Afghanistan, which underscores ongoing training of healthcare workers to ensure consistent SSC practices (34). ”

**References: Please review the reference and set them as per journal styles. References 1 and 4 are incomplete. Please provide availability link and access date.**

**Response:** Thank you for this suggestion. We have carefully reviewed and reformatted all references according to the journal’s style guidelines. In particular, references 1 and 4 have been completed with the required access links and retrieval dates.

**I noticed color changes in the text. Please use black color for all text.**

**Response:** Thank you for pointing this out. We have revised the manuscript to use black text throughout and ensured all text now consistently appears in black.
