# Supplementary material for: The role of implementation climate in shaping early essential newborn care practice: Insights from a multi-center cross-sectional study in China
Source: PLoS One. 2025 Oct 17;20(10):e0334855. doi: 10.1371/journal.pone.0334855 (PMC12533878; doi:10.1371/journal.pone.0334855)
Supplement: Attachment — Submitted filename: Response_to_Reviewers_auresp_2.docx [file pone.0334855.s006.docx]

**Response letter**

Dear Editors and Reviewers:

Thank you for your letter and for the time you have taken to review our manuscript. We greatly appreciate your valuable feedback, which has been essential in improving our paper. We have carefully studied all comments and have incorporated revision accordingly. The main corrections and our point-by point response are provided below.

**Journal Requirements:**

**1. If the reviewer comments include a recommendation to cite specific previously published works, please review and evaluate these publications to determine whether they are relevant and should be cited. There is no requirement to cite these works unless the editor has indicated otherwise.**

**Response:** Yes, we received a recommendation to cite a specific published study. We have reviewed the suggested paper, which examines the prevalence and factors associated with mother-newborn skin-to-skin contact in Afghanistan, and found it highly relevant to our work--particularly in supporting our findings related to training. Accordingly, we have cited this reference in the revised manuscript (lines 371-373).

**2. Please review your reference list to ensure that it is complete and correct. If you have cited papers that have been retracted, please include the rationale for doing so in the manuscript text, or remove these references and replace them with relevant current references. Any changes to the reference list should be mentioned in the rebuttal letter that accompanies your revised manuscript. If you need to cite a retracted article, indicate the article’s retracted status in the References list and also include a citation and full reference for the retraction notice.**

**Response:** We thank the editor for highlighting this important step. We have thoroughly reviewed all references in our manuscript and confirmed that none have been retracted. We have reviewed the reference list to ensure completeness and accuracy.

**Additional Editor Comments:**

**1. For PLOS ONE publication, we use [] for in-text citation. Please revise across the documents.**

**Response:** Thank you for pointing this out. We have revised all in-text citations throughout the manuscript to use square brackets in accordance with PLOS ONE style requirements.

**2. Line 301: Please put figure legend below the figure. Please upload Figure 1 as Per PLOS One style using https://ngplosjournals.pagemajik.ai/artanalysis. Delete figure 1 from document and upload as figure file after following journal guidelines. Only leave figure legend in the manuscript document.**

**Response:** Thank you for this guidance. We have removed Figure 1 from the manuscript document and uploaded it as a separate figure file following the journal’s guidelines.

3. **Tables 3 and 4. There is no single space between frequency and percentage brackets in some rows. Be consistent.**

**Response:** Thank you for this meticulous feedback. We have carefully reformatted Tables 3 and 4 to ensure consistent single spacing between frequency values and percentage brackets throughout.

**4. In tables 3 and 4, some cells have value less than 5, which increases the chances of uncertainty. It is better to correct or state as a limitation of the study.**

**Response:** We appreciate this suggestion. We have stated this as a limitation of the study in the revised manuscript (line 456-463).

**5. The term ‘early essential newborn care (EENC)’ was used in the manuscript several times. Once a term is abbreviated for the first time, it is not necessary to use the abbreviated form with its full form repeatedly. I can see the term is abbreviated in lines 226, 233, 307, and 404. Only the abbreviated term EENC can be used subsequently across the document.**

**Response:** Thank you for pointing this out. We have revised the manuscript to use the abbreviated form “EENC” after its first full introduction. The full term with abbreviation now appears only once, and the abbreviated form is used consistently thereafter.
